# Antigen-Specific Memory B-cell Responses to Enterotoxigenic *Escherichia coli* Infection in Bangladeshi Adults

**DOI:** 10.1371/journal.pntd.0002822

**Published:** 2014-04-24

**Authors:** Mohammad Murshid Alam, Amena Aktar, Sadia Afrin, Mohammad Arif Rahman, Sarmin Aktar, Taher Uddin, M. Arifur Rahman, Deena Al Mahbuba, Fahima Chowdhury, Ashraful Islam Khan, Taufiqur Rahman Bhuiyan, Yasmin Ara Begum, Edward T. Ryan, Stephen B. Calderwood, Ann-Mari Svennerholm, Firdausi Qadri

**Affiliations:** 1 Centre for Vaccine Sciences, International Centre for Diarrhoeal Disease Research, Bangladesh (icddr,b), Dhaka, Bangladesh; 2 Division of Infectious Diseases, Massachusetts General Hospital, Boston, Massachusetts, United States of America; 3 Department of Medicine, Harvard Medical School, Boston, Massachusetts, United States of America; 4 Department of Microbiology and Immunobiology, Harvard Medical School, Boston, Massachusetts, United States of America; 5 Department of Immunology and Infectious Diseases, Harvard School of Public Health, Boston, Massachusetts, United States of America; 6 Gothenburg University Vaccine Research Institute (GUVAX), Department of Microbiology and Immunology, University of Gothenburg, Gothenburg, Sweden; Christian Medical College, India

## Abstract

**Background:**

Multiple infections with diverse enterotoxigenic *E. coli* (ETEC) strains lead to broad spectrum protection against ETEC diarrhea. However, the precise mechanism of protection against ETEC infection is still unknown. Therefore, memory B cell responses and affinity maturation of antibodies to the specific ETEC antigens might be important to understand the mechanism of protection.

**Methodology:**

In this study, we investigated the heat labile toxin B subunit (LTB) and colonization factor antigens (CFA/I and CS6) specific IgA and IgG memory B cell responses in Bangladeshi adults (n = 52) who were infected with ETEC. We also investigated the avidity of IgA and IgG antibodies that developed after infection to these antigens.

**Principal Findings:**

Patients infected with ETEC expressing LT or LT+heat stable toxin (ST) and CFA/I group or CS6 colonization factors developed LTB, CFA/I or CS6 specific memory B cell responses at day 30 after infection. Similarly, these patients developed high avidity IgA and IgG antibodies to LTB, CFA/I or CS6 at day 7 that remained significantly elevated at day 30 when compared to the avidity of these specific antibodies at the acute stage of infection (day 2). The memory B cell responses, antibody avidity and other immune responses to CFA/I not only developed in patients infected with ETEC expressing CFA/I but also in those infected with ETEC expressing CFA/I cross-reacting epitopes. We also detected a significant positive correlation of LTB, CFA/I and CS6 specific memory B cell responses with the corresponding increase in antibody avidity.

**Conclusion:**

This study demonstrates that natural infection with ETEC induces memory B cells and high avidity antibodies to LTB and colonization factor CFA/I and CS6 antigens that could mediate anamnestic responses on re-exposure to ETEC and may help in understanding the requirements to design an effective vaccination strategies.

## Author Summary

Enterotoxigenic *Escherichia coli* (ETEC) is a non-invasive pathogen causing diarrhea in children as well as in adults and travelers in developing countries. After colonizing the intestine using colonization factors, the organisms secrete heat-stable (ST) and/or heat-labile (LT) enterotoxin to cause watery diarrhea. Natural infection with ETEC provides protection against subsequent infection; however, the precise mechanism is unknown. In this study, we have shown that adult patients with diarrhea infected with ETEC develop toxin (LTB) and colonization factor (CFA/I and CS6) specific memory B cell responses as well as highly avid antigen-specific antibodies. The antibody avidity indices were shown to be positively associated with memory B cell responses, suggesting that these processes may occur in concert. This study encourages further evaluation of such responses in children as well as in vaccinees.

## Introduction

Enterotoxigenic *Escherichia coli* (ETEC) are one of the major causes of diarrhea in developing countries, causing approximately 200 million episodes of diarrhea and 380,000 deaths every year [1], [2]. ETEC cause diarrhea in children less than 5 years of age in ETEC endemic areas as well as in adults and travelers to these areas [3], [4]. In recent years, ETEC have been shown to be the second most frequently isolated bacterial pathogens after *Vibrio cholerae* O1 in patients with diarrhea in Dhaka, Bangladesh [5]. ETEC strains are genetically and phenotypically divergent, expressing different toxins and one or more of several different colonization factors (CFs). The CFs facilitates the attachment of the bacteria to specific receptors on the intestinal mucosa [3], [6]–[8]. Production of the heat-stable (ST) and/or heat-labile (LT) enterotoxins then lead to a secretory diarrhea [9]. The LT toxin is a structural homolog and acts in a similar way as cholera toxin (CT) of *V. cholerae*, whereas the ST toxin is a nonimmunogenic small protein molecule [3], [9].

Protective immunity against ETEC infection is largely mediated by immune responses to CFs [10]–[14]. To date, more than 23 different CFs have been characterized and subdivided into different groups [3], [6], [15]. CFs with significant amino acid sequence similarity and cross-reactive epitopes are grouped together [6], [12]. The colonization factor-I (CFA/I) group is one of the predominant groups of CFs that has an important role in pathogenesis of human ETEC. This group includes CFA/I, CS1, CS2, CS4, CS14, CS17, CS19 and PCFO71 [5], [6], [16]–[18]. Human and animal studies have shown that antibodies to the fimbrial CFA/I cross-react immunologically with the other CFs of the CFA/I group [19]–[21]. Therefore, natural infection or vaccination with these related CFs might give protection against a wide variety of ETEC strains expressing CFs with cross-reactive epitopes. The non-fimbrial colonization factor CS6 has also been found to be one of the increasingly prevalent CFs present in ETEC strains in Bangladesh as well as in other ETEC endemic countries [5], [22], [23]. CS6 is expressed either alone or in association with CS4 or CS5 [6], and can produce both systemic and mucosal antibody responses following infection in children and adults [22]. CS6 also has been investigated as a vaccine antigen in human and animal studies using different routes of administration and formulations of antigen [22],[24]–[26]. Despite these different evaluations of immunity following ETEC infection and vaccination, long-term immune responses such as memory B cell responses and increases in antibody avidity to key ETEC antigens have not been previously described.

In this study, we have evaluated the generation of memory B cell responses and increases in antibody avidity to the T cell-dependent antigens LTB, CFA/I and CS6 following natural infection with ETEC in adult Bangladeshi individuals. We have also investigated IgA and IgG antibody responses in plasma, and circulation of antibody secreting cells (ASC) specific for these antigens after infection.

## Materials and Methods

### Study design and subject enrollment

We enrolled 52 adult patients admitted to the International Centre for Diarrheal Disease Research, Bangladesh (icddr,b) hospital with acute watery diarrhea between January 2008 and April 2013. Patients positive for ETEC but negative for *V. cholerae* O1 or O139, *Shigella*, *Salmonella* and other parasites tested were included in this study. Blood was collected from the patients at the time of enrollment (day 2) and again at day 7 and 30 post infection. At each time point, we assessed antigen (LTB, CFA/I and CS6)-specific antibody secreting cell (ASC), antibody responses and avidity of plasma antibodies to these antigens. Antigen-specific IgG and IgA memory B cell responses were measured on day 2 and 30.

### Ethics statement

Prior to enrollment, written informed consent was obtained from patients. The study was approved by the Research Review and Ethical Review Committees of the icddr,b, Dhaka, Bangladesh.

### Isolation of peripheral blood mononuclear cells (PBMC)

Sodium-heparinized blood was diluted two-fold with phosphate-buffered saline (PBS, pH 7.2–7.4) and peripheral blood mononuclear cells (PBMCs), and plasma samples were isolated after centrifugation on Ficoll-Isopaque (Pharmacia, Piscataway, NJ) [27]. Plasma was stored at −20°C for further immunological assays. PBMCs were washed and resuspended at a concentration of 10^7^ cells/ml in RPMI complete medium (Gibco, Carlsbad, CA) with 10% heat-inactivated fetal bovine serum (HyClone, Logan, UT) [27]. The cells were then immediately used for the enzyme-linked immunosorbent spot (ELISPOT) assay to determine ASC responses, or cells were cultured for memory B cell responses as described below.

### Detection of LTB- and CF-specific antibody responses in plasma

CFA/I-, CS6- and LTB-specific IgA and IgG responses in plasma were assessed by using standardized enzyme-linked immunosorbent assay (ELISA) technique as described previously [28]. Briefly, 96-well polystyrene plates (Nunc F, Denmark) were coated with GM1 ganglioside (0.3 nM; University of Gothenburg, Sweden) overnight followed by recombinant cholera toxin B subunit (CTB) (0.5 µg/ml; University of Gothenburg, Sweden), or with ETEC colonization factors CFA/I (University of Gothenburg) or CS6 (1 µg/ml; gift from F Cassels, Walter Reed Army Institute of Research, Maryland). LTB has 80% nucleotide sequence similarity to CTB, and cross- reacts immunologically with CTB [15], [29]. Therefore, we used CTB as the antigen to detect responses to LTB. For detecting each antigen-specific antibody, 150 µl/well of plasma (initially diluted in 0.1% bovine serum albumin [BSA] in PBS- 0.05%Tween; 1∶100 for LTB and 1∶10 for CFA/I and CS6) were added, serially 3-fold diluted. The presence of antigen-specific antibodies were detected using horseradish peroxidase (HRP)-conjugated anti-human IgA and IgG, (Jackson ImmunoResearch, West Grove, PA; 1∶2000 dilution in 0.1% BSA-PBS-Tween) and ortho phenylenediamine (Sigma, St. Louis, MO) in 0.1 M sodium citrate buffer (pH 4.5) and hydrogen peroxide. The reactions were stopped after 20 minutes by adding 1 M H_2_SO_4_ and endpoint titers were measured as the reciprocal interpolated dilutions of the samples at 492 nm that were 0.4 above background. ELISA data were normalized by calculating the ratio of the test sample to a standard of pooled sera derived from the convalescent phase of ETEC-infected patients used as a positive control on each plate. These normalized values were multiplied by 100 and defined as ELISA units. Patients with a ≥2 fold increase in the anti-LTB, CFA/I and CS6 antibody responses were considered as responders.

### Measurement of LTB- and CF-specific antibody avidity

CFA/I-, CS6- and LTB- specific IgA and IgG antibody avidity indices were assessed using previously described methods [30], [31]. ELISA was carried out to assess CFA/I-, CS6- and LTB- specific IgA and IgG antibody responses as described above. After completion of incubation of the plasma (1∶3 to 1∶1500 dilution in 0.1% BSA-PBS-Tween) in wells coated with antigens, half of the total wells were treated with sodium thiocyanate (NaSCN) (2 M NaSCN in PBS–0.3% Tween), whereas the remaining half of wells were treated with PBS–0.3% Tween alone for 20 min at room temperature. Horseradish peroxidase-conjugated anti-human IgA or IgG antibodies (Jackson Laboratories, Bar Harbor, ME) were used at a 1∶1,000 dilution to determine CFA/I-, CS6- and LTB-specific IgA or IgG responses, as described above. For each sample, an OD result between 0.5 to 2.0 in the non-NaSCN wells was used for the calculation of antibody avidity. The avidity index was calculated as the ratio of the OD of NaSCN treated wells to the OD of the untreated wells as described previously [30], [31]. Pooled sera derived from patients in the convalescent phase of ETEC infection was used as a positive control on each plate.

### Determination of circulating ASC

Antigen-specific IgA and IgG ASC responses were determined using ELISPOT, as previously described [12], [22], [32]. Briefly, nitrocellulose-bottomed plates (MSHAN4550; Millipore, Bedford, MA) were coated with CFA/I (10 µg/ml), CS6 (10 µg/ml), or GM1 ganglioside (3 nM) followed by recombinant CTB (2.5 µg/ml). We also coated plates with affinity-purified goat anti-human immunoglobulin (5 µg/ml; Jackson Immunology Research, West Grove, PA) to measure total IgA and IgG antibody secreting cells, or with keyhole limpet hemocyanin (KLH, 2.5 µg/ml; Pierce Biotechnology, Rockford, IL) as a negative control. Antigen-specific ASCs were detected using alkaline phosphatase-conjugated goat anti-human IgG (Southern Biotech, Birmingham, AL) and horseradish peroxidase -conjugated IgA (Southern Biotech, Birmingham, AL), followed by 5-bromo-4-chloro-3-indolylphosphate–nitroblue tetrazolium (BCIP-NBT; Sigma, St. Louis, MO) and 3-amino-9-ethyl carbazole (AEC), respectively. Independent counts visualized by two individuals using a stereomicroscope (red spots indicating IgA and blue spots indicating IgG on the same wells) were averaged, and results were expressed as percentage of antigen-specific ASC out of total ASC of that isotype.

### Memory B cell culture and assay

Memory B cells were assessed on days 2 and 30 by an ELISPOT technique, as previously described [27], [33], [34]. Freshly isolated PBMCs from blood were cultured in 24-well cell culture plates (BD Biosciences, San Jose, CA) at 5×10^5^ PBMCs/well in RPMI 1640 medium containing 10% FBS, 2 mM L-glutamine, 200 units/ml penicillin, 200 µg/ml streptomycin, and 50 µM beta-mercaptoethanol, along with a mixture of three B-cell mitogens including CpG oligonucleotide (6 µg/ml; Operon, Huntsville, AL), a 1/100,000 dilution of crude pokeweed mitogen extract, and a 1/10,000 dilution of fixed *Staphylococcus aureus* Cowan (Sigma, St. Louis, MO). As a negative control, cells were placed in wells without the mitogen mixture. After incubating the cells at 37°C with 5% CO_2_ for 6 days, cells were harvested and memory B cell responses were determined by examining for ASC that had matured under stimulation using an ELISPOT method as described above. We detected memory B cell responses using horseradish peroxidase-conjugated mouse anti-human IgA and IgG (Hybridoma Reagent Laboratory, Baltimore, MD), followed by AEC. Antigen-specific memory B cell responses were expressed as the percentage of antigen-specific ELISPOT counts out of the total isotype-specific Ig-reactive cells. The limits of detection for responders for antigen-specific IgA and IgG was defined as 0.004% and 0.001%, respectively, per 5×10^5^ PBMC after 6 days of stimulation [27], [33]. The inclusion and exclusion criteria for analyses of data were used as previously described [35].

### Statistical analyses

Statistical analyses were performed using GraphPad Prism 4 (GraphPad Software, Inc., La Jolla, CA). Immunological responses were compared within groups for significance using Wilcoxon signed-rank test. We used Spearman's correlation analysis to measure bivariate associations. All reported *P* values were two tailed and statistical significance was defined as a *P* value of ≤0.05.

## Results

### Study population

In this study, we enrolled 52 adult (median age 32 years, 48% female) patients with diarrhea who were infected with ETEC. Of the patients, 60%, 19% and 21% were infected with LT/ST-, LT- or ST- expressing ETEC respectively. The majority of the patients were infected with ETEC expressing either CFA/I group (n = 15, 29%) or CS6 with or without CS5 (n = 12, 23%) as their colonization factor. Most of the ETEC strains expressing CFs cross-reacting with CFA/I, or CS6 with or without CS5, produced either LT or both LT and ST (73 and 75%, respectively), whereas all 4 strains expressing CFA/I itself produced ST only. Eighty seven percent of the patients completed follow up to day 30. The demographic, clinical and microbiological characteristics are presented in Table 1.

**Table 1:** Demographic, clinical and microbiological characteristics of patients.

Characteristics	
Number of study participants	52
Follow up completed to day 30 (%)	45 (87%)
Median age in yr (25^th^, 75^th^ percentile)	32 (25,40)
Gender, female (%)	25 (48%)
Mean (SD) duration of diarrhea prior to hospitalization, hours	21.66 (18.98)
Mean (SD) duration of hospital stay, hours	32.19 (22.92)
Toxin types of ETEC strains, n (%)	
LT/ST	31 (60%)
LT	10 (19%)
ST	11 (21%)
Colonization factors of ETEC strains, n	
CFA/I group strains: (CFA/I, CS17, CS4, CS14)	10 (3, 2, 1, 4)
CFA/I group CS1+CS3+CS21, CS2+CS3, CFA/I+CS21	5 (3, 1, 1)
CS6 (CS6 only, CS5+CS6)	12 (6, 6)
CS7	2
CF negative	23

### Heat labile toxin B subunit (LTB) and colonization factor (CF) specific antibody responses in plasma

**Figure 1:**
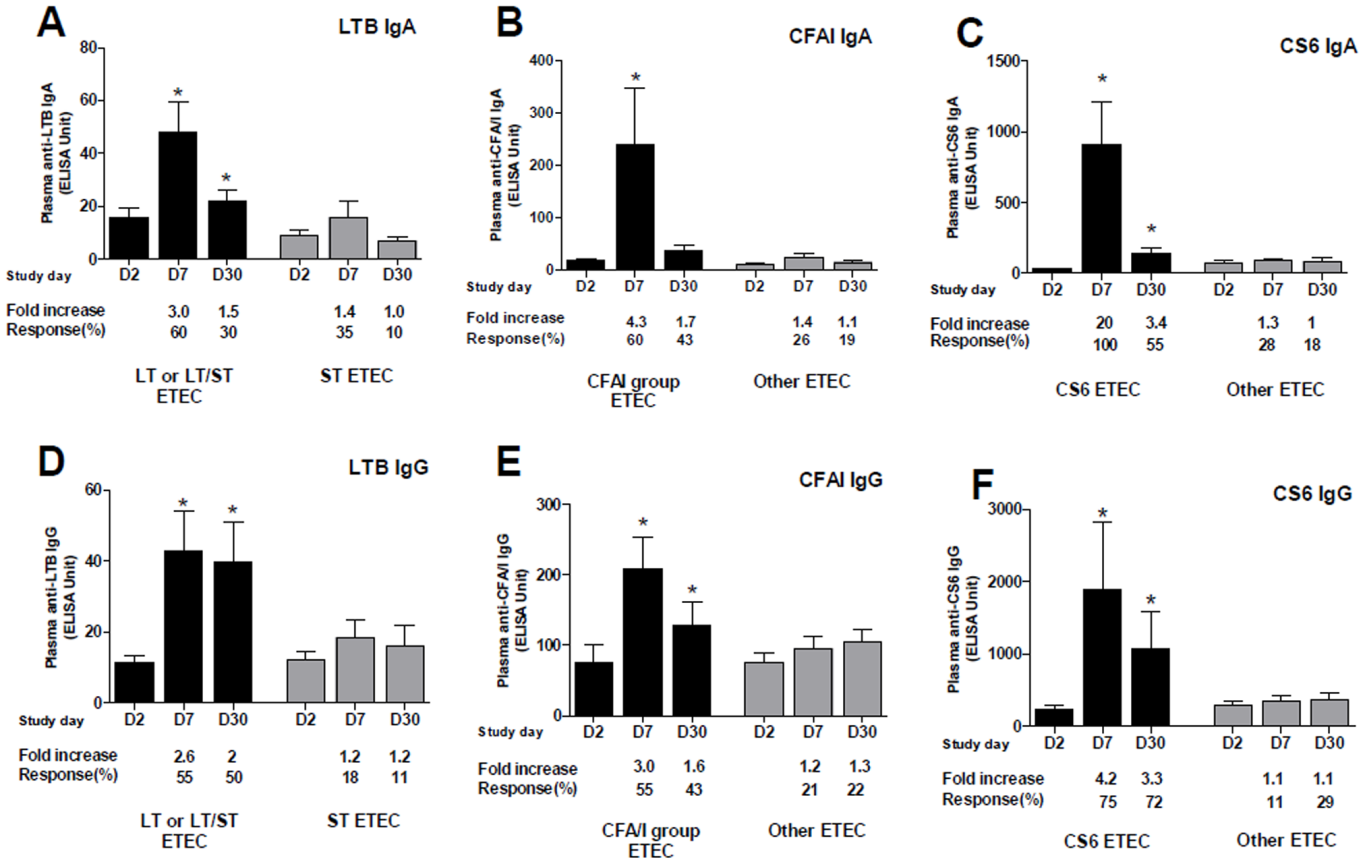
Anti-LTB (A and D), CFA/I (B and E) and CS6 (C and F) specific IgA and IgG antibody responses in plasma in Bangladeshi adults infected with ETEC. The columns indicate mean responses and the error bars represent standard errors of the mean (SEM). An asterisk denotes a statistically significant difference (*P*<0.05) from the acute stage of infection (day 2). Mean fold changes and responder frequencies compared to day 2 levels are also indicated.

LTB and colonization factor CFA/I or CS6 specific IgA and IgG antibody responses in plasma were measured at the acute stage of infection (day 2) and again on days 7 and 30. Statistically significant increases of IgA and IgG antibody responses to LTB were observed on day 7 when compared to responses seen at the acute stage (*P*<0.0001 for both isotypes) in patients infected with ETEC expressing LT or LT/ST (Fig. 1A and D). The LTB-IgA and IgG antibody levels remained elevated up to day 30 compared to day 2 results (*P*<0.003 for both isotypes). We did not detect any response to LTB in patients infected with ETEC expressing ST alone. CFA/I-specific IgA and IgG antibody responses also peaked on day 7 in patients infected with ETEC expressing CFA/I group CFs (*P*<0.002) (Fig. 1B and E). Of note, patients infected either with homologous (response rates, 75% for IgG and 100% for IgA) or cross-reacting (response rate, 50% for both IgG and IgA) CFs responded well with IgG and IgA antibodies to CFA/I. The IgG responses to CFA/I remained elevated up to day 30 (*P* = 0.004), whereas the IgA responses were short-lived and had decreased to baseline by day 30. We did not see significant IgA or IgG antibody responses to CFA/I in patients infected with ETEC expressing other CFs or no CFs. Similarly, patients infected with ETEC expressing CS6, with or without CS5, showed significant increases in CS6 IgA and IgG antibody responses at day 7 (*P*<0.004) (Fig. 1C and F). Both IgA and IgG antibody levels remained elevated up to day 30 when compared with day 2 (*P*<0.02). Responses against CS6 were not observed in patients infected with ETEC expressing CFs other than CS6.

### Heat labile toxin B subunit (LTB) and colonization factor (CF)-specific antibody avidity in plasma

**Figure 2:**
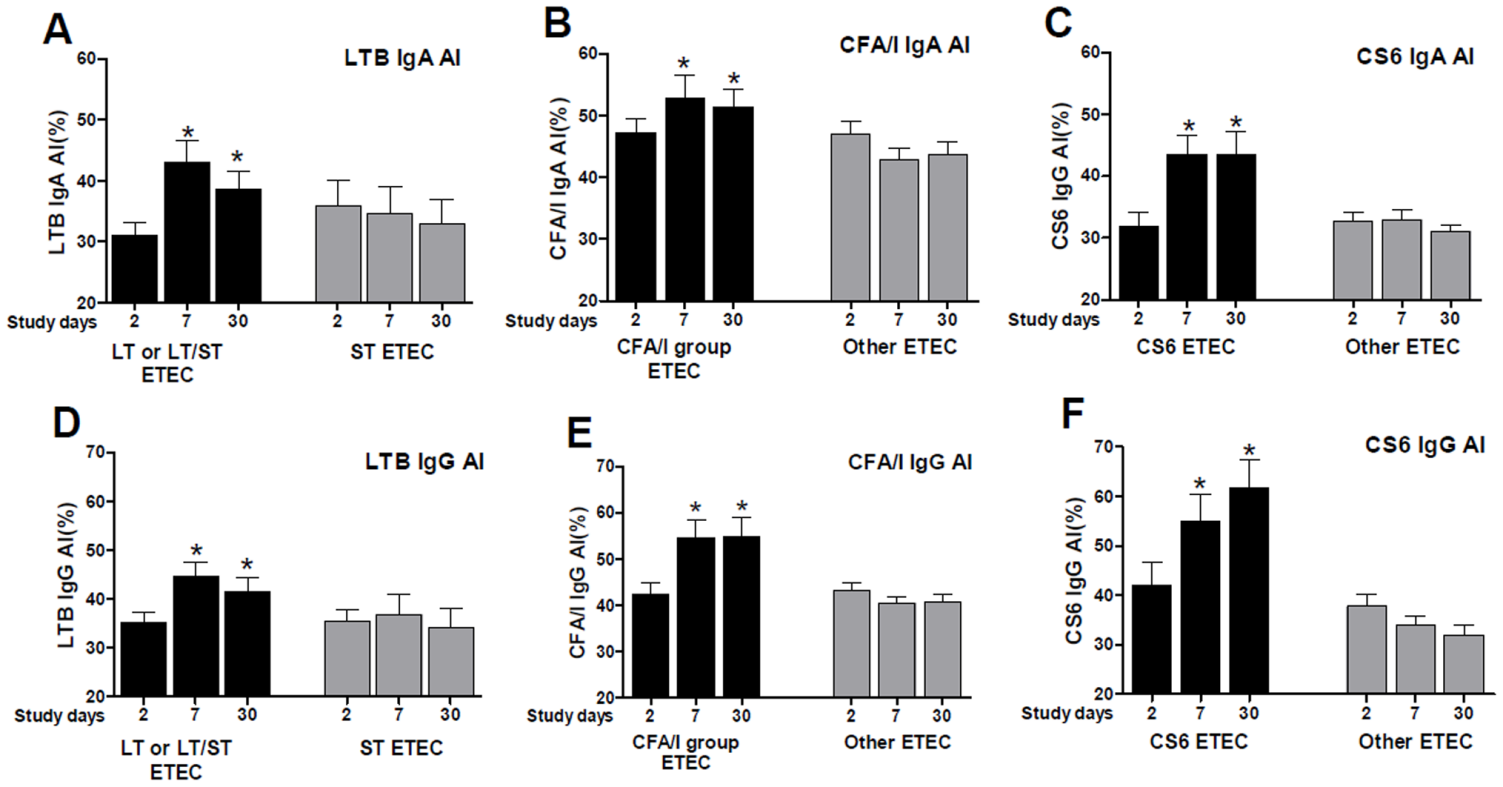
Avidity indices of LTB (A and D), CFA/I (B and E) and CS6 (C and F) specific IgA and IgG antibodies in plasma in Bangladeshi adults infected with ETEC. Columns indicate mean avidity indices, and error bars represent standard errors of the mean (SEM). An asterisk denotes a statistically significant difference (*P*<0.05) from the acute stage of infection (day 2).

The mean avidity index (AI) for IgA and IgG antibodies to LTB at day 2 was 31% and 35%, respectively (Fig. 2A, 2D). These increased significantly at day 7 (AI = 43% and 45%, respectively) (*P*<0.003) and remained elevated up to day 30 (AI = 39% and 41%, respectively) (*P*<0.05) in patients infected with ETEC expressing LT or LT/ST. Significant increases in AI of CFA/I-specific IgA and IgG antibodies was also observed (Fig. 2B,2E) the increase in AI was more robust for IgG antibodies than IgA. The AI remained elevated up to day 30, with AI of 51% and 55%, respectively (*P*<0.05). Similarly, patients infected with ETEC expressing CS6 with or without CS5 developed high avidity IgA and IgG antibodies to CS6 at day 7 (AI = 44% and 55%, respectively) when compared to day 2 (AI = 32% and 42%, respectively) (*P*<0.007), (Fig. 2C,2E) and the AI remained elevated at day 30 (AI = 43% and 61%, respectively) for both isotypes of antibodies (*P*<0.05).

### Antigen specific IgA and IgG ASC responses

**Figure 3:**
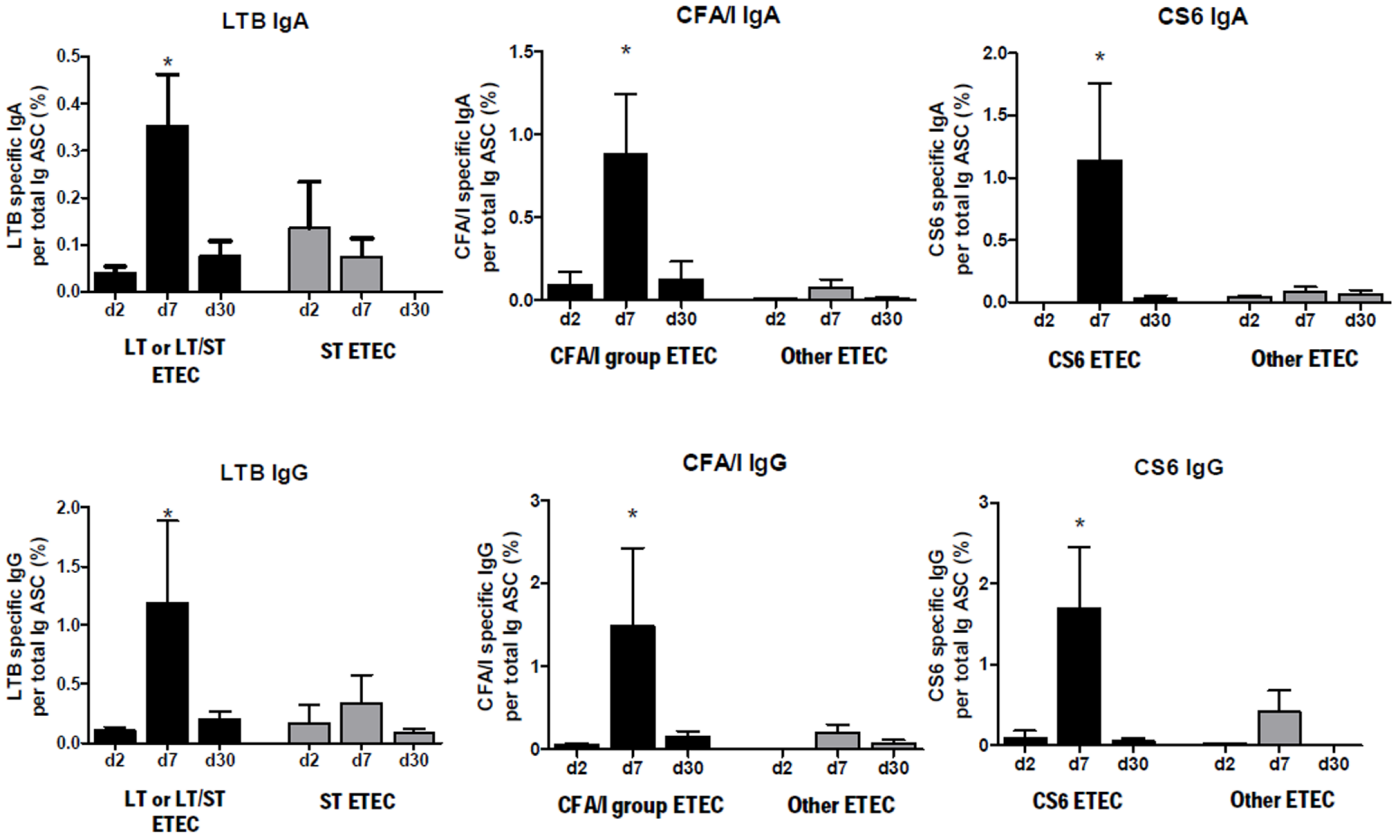
Mean + standard errors of the mean (SEM) circulating IgA/total IgA and IgG/total IgG ASC responses to LTB, CFA/I and CS6 in adult Bangladeshi ETEC patients. An asterisk denotes a statistically significant difference (*P*<0.05) from the acute stage of infection (day 2).

LTB, CFA/I and CS6-specific IgA and IgG specific ASC responses were measured on days 2, 7 and 30. As expected, ASC responses to all three antigens peaked at day 7 and had waned at day 30 in patients infected with ETEC expressing LT or LT/ST, CFA/I group CFs or CS6, respectively (Fig. 3) (*P*<0.05). We also observed that the response rate in patients infected with CFA/I (response rate, 75% for IgA and IgG) or CFA/I cross-reactive CFs (response rate, 70–80% for IgA and IgG) were comparable. However, we did not detect such responses in patients infected with ETEC expressing other or no known CFs.

### Antigen-specific memory B cell responses

**Figure 4:**
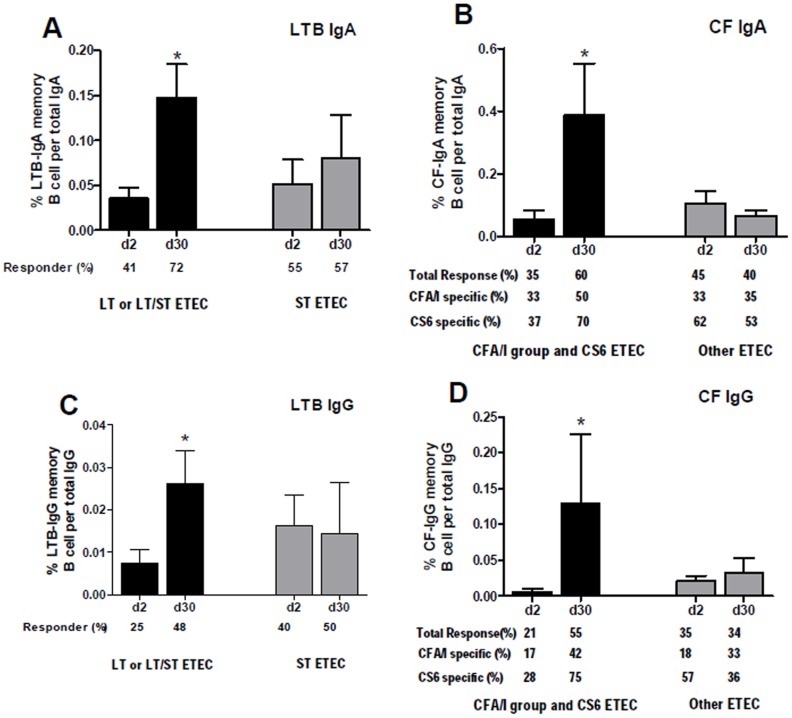
LTB (A and C) and CF (B and D) specific memory B cell responses in patients infected with ETEC. The columns indicate mean responses and the error bars represent standard errors of the mean. An asterisk denotes a statistically significant difference (*P*<0.05) from the acute stage of infection (day 2).

Memory IgA and IgG B cell responses to LTB, CFA/I and CS6 were measured on days 2 and 30. Significant LTB-specific IgA and IgG memory B cell responses were observed on day 30 in patients infected with ETEC expressing LT or LT/ST compared to day 2 (*P*<0.04) (Fig. 4A and C). Detectable IgA and IgG memory B cell responses to LTB were found in 41% and 25% of patients at the acute stage of infection, and that increased to 72% and 48%, respectively, at day 30 in LT or LT/ST infected patients. However, we did not detect memory B cell responses to LTB in patients infected with ST-expressing ETEC. We also measured memory B cell responses to colonization factors CFA/I and CS6, combining data from patients infected with ETEC expressing CFA/I group CFs or CS6 due to paucity of matched paired data for statistical analyses. We detected a significant increase of CF (CFA/I or CS6)-specific IgA and IgG memory B cell responses at day 30 when compared to the memory B cell responses at day 2 (P≤0.05) (Fig. 4B and D). Detectable CFs (CFA/I and CS6)-specific IgA and IgG memory B cell responses were observed in 35% and 21% of patients, respectively, at day 2, and that increased to 60% and 55%, respectively, at day 30. CFA/I specific memory B cell responses were not only observed in CFA/I infected patients, but also in patients infected with strains expressing cross-reacting CFs. Detectable CFA/I-specific IgA and IgG memory B cell responses were found in 50–65% of the patents infected with strains expressing CFA/I). However, we did not detect CFA/I or CS6-specific memory B cell responses in patients infected with CF negative ETEC or ETEC expressing heterologous CFs.

### Correlation between development of antigen-specific memory B cell responses and increased antibody avidity

To determine potential correlation between development of antigen-specific memory B cell responses and increased antibody avidity, we compared LTB, CFA/I and CS6-specific IgA and IgG memory B cell responses with avidity indices on corresponding study days in patients infected with ETEC expressing LT or LT/ST, CFA/I or CS6 antigen, respectively. For all three antigens, we detected a positive correlation between development of memory B cell responses and increases of avidity indices of IgA and IgG antibodies on corresponding study days (Spearman, r = 0.32 to 0.6, *P* = 0.003–0.1), although correlation for IgG responses to CS6 did not reach statistical significance and correlation for LTB specific responses were weak (Spearman, r = 0.323 to 0.338, *P* = 0.026–0.054) (Table 2).

**Table 2:** Correlation analyses* of antibody avidity index (AI) and memory B cell (MBC) responses in patients infected with ETEC.

Correlations between different groups	Spearman r	*P*-value
LTB specific		
IgG-AI and IgG-MBC	0.338	0.026
IgA-AI and IgA- MBC	0.323	0.054
CFA/I specific		
IgG-AI and IgG- MBC	0.437	0.047
IgA-AI and IgA- MBC	0.601	0.003
CS6 specific		
IgG-AI and IgG- MBC	0.437	0.103
IgA-AI and IgA- MBC	0.574	0.020

*Spearman's test was used for correlation analyses.

## Discussion

In this study, we have shown for the first time the development of memory B cell responses as well as increased avidity antibodies to homologous and cross-reacting ETEC antigens in hospitalized patients with ETEC diarrhea. The development of memory B cells and high avidity IgA and IgG antibodies to CFA/I or CS6 were detected in patients infected with CFA/I group or CS6 ETEC, respectively, but not in patients infected with ETEC expressing heterologous CFs. LTB specific responses were only observed in patients infected with ETEC expressing LT or LT/ST, but not with ST-only expressing ETEC. Similar patterns of ASC and antibody responses were also observed to CFA/I, CS6 and LTB in patients infected with homologous or cross-reacting antigens. We also show that there was a correlation between development of increased antibody avidity and development of memory B cell responses to the same antigen on corresponding study days, suggesting that these responses may occur simultaneously as part of maturation of B cells and antibody maturation during somatic hypermutation following antigenic stimulation.

Memory B cells are thought to play a critical role in eliciting a rapid anamnestic antibody response upon antigen re-exposure, and thus to play a key role in mediating long-lasting immunity [34], [36]. During a primary infection, presence of an antigen at the mucosal surface generates a cascade of interactions in the germinal center (GC) of the secondary lymphoid organs [37]. T-cell dependent protein antigens induce differentiation of CD4+ helper T cells into follicular helper T cells (T_FH_) that subsequently interact with B cells in the GC and stimulate B cell proliferation, somatic hypermutation, and isotype switching. These events in the GC lead to generation of memory B cells as well as plasma cells producing antibodies with increased avidity [37], [38], and both of these have been suggested to play a critical role in protective immunity. We have shown that in *V. cholerae* O1 infection, development of memory B cell responses and high avidity antibodies to T cell dependent protein antigens develop and persist over the course of a year following infection [27], [30]. Similar measurement of memory B cell responses and antibody avidity to key protein antigens following ETEC infection has not previously been reported.

Protective immunity against ETEC is most likely dependent on mucosal immunity, with secretion of antibodies that prevent binding of bacteria and/or toxins to mucosal epithelial cells [39], [40]. Human and animal studies have shown that antibodies to CF antigens provide protection against infection with ETEC expressing the homologous CFs [11], [13], [14], [41], [42]. CFA/I can prime and boost antibody responses, as well as ASC responses, against the cross reacting CS4 antigen and vice versa [43]. Adult Bangladeshi patients infected with CFA/I expressing ETEC also respond with significant IgA antibody responses to the cross reactive CS1, CS2, CS4, CS14 and PCFO166 colonization factors, in addition to the homologous CFA/I antigen [12], [21]. This suggests that ETEC can stimulate immune responses to both homologous and cross reacting CFs. In concordance with the above findings, we here report that patients infected with ETEC expressing CFA/I group CFs other than CFA/I develop anti-CFA/I-IgG and IgA ASC and antibody responses. Moreover, these patients develop memory B cell responses and highly avid IgA and IgG antibodies to CFA/I, suggesting that immune responses against cross-reactive epitopes may be protective. We have previously shown that mucosal and systemic immune responses also develop in humans infected with ETEC expressing CS6, with or without CS4 or CS5 [22], and in recent studies, that oral immunization of mice with inactivated *E. coli* bacteria overexpressing CS6 induces substantial immune responses against this antigen [25]. In this study, we show that patients infected with CS6-expressing ETEC not only develop CS6-specific IgG and IgA ASC and antibody responses, but also memory B cell responses and highly avid anti-CS6 IgA and IgG antibodies after onset of diarrhea.

Several human and animal studies have shown that both LT and CT immunity can provide protection against ETEC strains expressing LT or LT/ST [26], [39]. LT can also act as a mucosal adjuvant to boost immune responses. In this study, we show the development of memory B cell responses and high avidity IgA and IgG antibodies to LTB, responses that may play a role in immunity following ETEC infection.

The present data on memory B cell responses and antibody avidity to different ETEC antigens following infection may provide insights into vaccine development. The multivalent nature of the antigens expressed by different ETEC strains makes design of a broadly effective vaccine challenging. Efforts have been made to develop live attenuated [44] as well as inactivated whole cell ETEC vaccines expressing high levels of the most prevalent CFs , i.e. CFA/I, CS1-CS6, which have the potential to also provide cross-reactive immunity against ETEC expressing related CFs [25], [40], [45]. These vaccines have been tested in human and animals and proven to be safe and provide strong mucosal immune responses [25], [44], (Lundgren et al, to be published). Similarly, fimbrial tip adhesions are being evaluated as vaccine candidates against ETEC infection [46]. Further studies of memory B cell responses and antibody avidity to key antigens after administration of these vaccines will be of interest.

In summary, our results show that adult patients with ETEC diarrhea develop toxin (LTB) and colonization factor (CFA/I and CS6) specific memory B cell responses, as well as highly avid IgG and IgA antibodies to these same antigens; development of these immune responses correlates with each other, consistent with the known pathways of B cell development and maturation. Patients infected with ETEC expressing the CFA/I group CFs not only develop immune responses to the homologous CFA/I antigen but also to cross-reactive CFs, suggesting that such infections may provide broad spectrum immunity. These findings encourage further studies to evaluate whether memory B cell responses, affinity maturation and level of antibodies to these ETEC antigens correlate with protection following natural infection.
